# 

**Published:** 1882-03

**Authors:** 


### *TABLE OF CONTENTS FOR MARCH, 1882.*

### CLINICAL LECTURES.

Art. 1.—Facial Paralysis. By Jas. T. Whittaker, M. D. . . . . . . . . . . . . . . . 145

### ORIGINAL COMMUNICATIONS.

Art. 2.—Non-Identity of Croup and Diphtheria. By T. S. Latimer, M. D...153
Art. 3.—Eight Cases of Syphilis of the Finger in Medical Men. By Fessenden N. Otis, M. D. . . . . . . . . . . . . . . . . . . . . . . . . . . . . . . . . . . . . . . . . . . . 158
Correspondence. . . . . . . . . . . . . . . . . . . . . . . . . . . . . . . . . . . . . . . . . . . . . . . . 163

### ORIGINAL TRANSLATIONS AND ABSTRACTS.

Value of Jaborandi and Pilocarpine in Eye Diseases. . . . . . . . . . . . . . . . . . . . . 164
Investigation into the Effects of Organisms upon the Teeth and Alveolar Portions of the Jaws. . . . . . . . . . . . . . . . . . . . . . . . . . . . . . . . . . . . . . . . . . 167
A new (Bloodless) Method of Removing Subserous Fibroids of the Uterus. .173
Buccal Ulcerations of Constitutional Origin. . . . . . . . . . . . . . . . . . . . . . . . . . . 174

### SELECTIONS.

Hints in Operating for Fistula in Ano. . . . . . . . . . . . . . . . . . . . . . . . . . . . . . 176
The Action and Uses of Pilocarpine. . . . . . . . . . . . . . . . . . . . . . . . . . . . . . . . 178
The Use of Plaster of Paris in Fractures. . . . . . . . . . . . . . . . . . . . . . . . . . . . . 180
Cerebral Excitement Caused by the Bromides. . . . . . . . . . . . . . . . . . . . . . . . . . 181
A New Method of Embalming bodies and preserving Tissues. . . . . . . . . . . . . .182
Viburnum Prunifolium in Uterine Diseases. . . . . . . . . . . . . . . . . . . . . . . . . . . . 183
Artificial Hunyadi Janos Water. . . . . . . . . . . . . . . . . . . . . . . . . . . . . . . . . . . . 184
Removal of the Kidneys for Nephro-Lithiasis. . . . . . . . . . . . . . . . . . . . . . . . . . 184
Aspiration of the Bowels in Peritonitis. . . . . . . . . . . . . . . . . . . . . . . . . . . . . . . 185
Prof. Pasteur and the Yellow Fever. . . . . . . . . . . . . . . . . . . . . . . . . . . . . . . . . 186
Some Facts About Diphtheria. . . . . . . . . . . . . . . . . . . . . . . . . . . . . . . . . . . . . . 186
Chronic General Peritonitis. . . . . . . . . . . . . . . . . . . . . . . . . . . . . . . . . . . . . . . 187
Resuscitation of Frozen Animals. . . . . . . . . . . . . . . . . . . . . . . . . . . . . . . . . . . . 187
Aconite Poisoning. Venereal Warts. . . . . . . . . . . . . . . . . . . . . . . . . . . . . . . . . 188
"Ethereal" Castor Oil. Effect of Drugs on Lactation. . . . . . . . . . . . . . . . . . . . . . 189
Improvements in Hypodermic Injection. . . . . . . . . . . . . . . . . . . . . . . . . . . . . . . 189
Treatment of Frequently Recurring "Erysipelas" of the Face. . . . . . . . . . . . . . 190
Giving Opium. . . . . . . . . . . . . . . . . . . . . . . . . . . . . . . . . . . . . . . . . . . . . . . . . . 190
Contagious Peripneumonia and Preventive Inoculation. . . . . . . . . . . . . . . . . . . 190
Osseus Lesions in Hemiplegia. . . . . . . . . . . . . . . . . . . . . . . . . . . . . . . . . . . . . 191
Scrotal Calculus. . . . . . . . . . . . . . . . . . . . . . . . . . . . . . . . . . . . . . . . . . . . . . . . 192
Effects of Excision of Syphilitic Chancre. . . . . . . . . . . . . . . . . . . . . . . . . . . . . . 192
Injections of Pilocarpine in Edema of the Glottis. . . . . . . . . . . . . . . . . . . . . . . . 193
When Does the Danger of Infection in Scarlatina Cease?. . . . . . . . . . . . . . . . . 193
Treatment of Tympanites. . . . . . . . . . . . . . . . . . . . . . . . . . . . . . . . . . . . . . . . . 193

### EDITORIAL DEPARTMENT.

The Crusade Against Physiological Experiment in England. . . . . . . . . . . . . . 194
The Senior Editor on His Rambles. . . . . . . . . . . . . . . . . . . . . . . . . . . . . . . . . . 196
Current News and Opinion . . . . . . . . . . . . . . . . . . . . . . . . . . . . . . . . . . . . 197–198
Obituary. . . . . . . . . . . . . . . . . . . . . . . . . . . . . . . . . . . . . . . . . . . . . . . . . . . . . . 199
Reviews and Bibliographical Notices. . . . . . . . . . . . . . . . . . . . . . . . . . . . . 200–202

### POPULAR SCIENCE DEPARTMENT.

The Relative Importance of Various Subjects in Practical Sanitation. . . . . . .203
Forms and Functions of the Electric Discharge. . . . . . . . . . . . . . . . . . . . . . . . . 210
Keeping Vegetables in Winter. . . . . . . . . . . . . . . . . . . . . . . . . . . . . . . . . . . . . 212
Copying Ink Without a Press. . . . . . . . . . . . . . . . . . . . . . . . . . . . . . . . . . . . . . 214
Gout and an Animal Diet. . . . . . . . . . . . . . . . . . . . . . . . . . . . . . . . . . . . . . . . . 215

